# Characteristic Pattern of IL-17RA, IL-17RB, and IL-17RC in Monocytes/Macrophages and Mast Cells From Patients With Bullous Pemphigoid

**DOI:** 10.3389/fimmu.2019.02107

**Published:** 2019-09-11

**Authors:** Stéphane Nesmond, Céline Muller, Richard Le Naour, Manuelle Viguier, Philippe Bernard, Frank Antonicelli, Sébastien Le Jan

**Affiliations:** ^1^Laboratory of Dermatology, EA7509 IRMAIC, University of Reims-Champagne-Ardenne, Reims, France; ^2^Department of Biological Sciences, UFR Pharmacy, University of Reims Champagne-Ardenne, Reims, France; ^3^Department of Dermatology, University Hospital, University of Reims-Champagne-Ardenne, Reims, France; ^4^Department of Biological Sciences, Immunology, UFR Odontology, University of Reims-Champagne-Ardenne, Reims, France

**Keywords:** autoimmunity, inflammation, bullous pemphigoid, IL-17 receptors, IL-17 isoforms

## Abstract

Inflammation is largely implicated in bullous pemphigoid (BP), the most frequent skin auto-immune blistering disease. IL-17, essentially IL-17A/F, has been involved in blister formation through regulation of protease production, and its specific serum profile within BP was related to disease outcome. However, relationships between IL-17 family ligands and receptors are quite complex with six different IL-17 isoforms, and five different receptors. We here aimed at clarifying the contribution of the IL-17 axis in BP by characterizing not only the expression of IL-17 receptor (IL-17R) members within immune cells isolated from BP patients (PMNs, *n* = 9; T-lymphocytes, *n* = 10; and monocytes, *n* = 10) but also the expression of IL-17 isoforms in sera (*n* = 83), and blister fluid (*n* = 31) of BP patients. We showed that at diagnosis, IL-17RA and IL-17RC expression were significantly increased in monocytes isolated from BP patients as compared to those from control subjects (*p* = 0.006 and *p* = 0.016, respectively). Notably, both IL-17RA and IL-17RC mRNA expression remained elevated in BP monocytes at time of relapse. We further demonstrated a significant increase of all IL-17 isoforms tested in BP blister fluid compared with BP serum (IL-17A, *p* < 0.0001; IL-17A/F, *p* < 0.0001; IL-17B, *p* = 0.0023; IL-17C, *p* = 0.0022; IL-17E, *p* < 0.0001). Among all, IL-17B was the only cytokine for which a significant decreased concentration within blister fluid was observed in BP patients with severe disease compared to patients with moderate disease (*p* = 0.012). We further evidenced a significant negative correlation between IL-17B levels and blister/erosion BPDAI subscore (*r* = −0.52, *p* = 0.003). We finally identified mast cells as a potential target of IL-17B in lesional skin of BP patients. In conclusion, we showed here that IL-17RA and IL-17RC expression in monocyte was associated with disease activity and evidenced *in situ* a negative correlation between BP disease activity and IL-17B, whose effects could be mediated by IL-17RB expressed by mast cell in BP lesional skin.

## Introduction

Bullous pemphigoid (BP) is the most frequent blistering skin disease of autoimmune origin and affects mainly the elderly (1–6). Clinical features of BP are intense pruritus associated with tense bullae, inflammatory erythematous plaques, and itching (1, 2, 6, 7). BP is characterized by the production of autoantibodies against two proteins of the hemidesmosome structure, BP180, and BP230 (8–13). The binding of BP autoantibodies onto their target induces blister formation by activating complement pathway, which triggered subsequent recruitment of inflammatory cells at the dermal–epidermal junction. Secretion of inflammatory key mediators by inflammatory cells leads up to an overexpression of proteases, such as the matrix metalloproteinase-9 (MMP-9) and the neutrophil elastase, which are involved in dermal–epidermal splitting (14–23). However, autoantibody level does not explain all clinical features of BP (24), suggesting that other regulatory mechanisms are involved in the inflammatory response associated with BP. In this line, we previously showed that IL-17 within the blister fluid (BF) of BP patients was related to MMP-9 production (25), and that specific patterns of IL-17 within the serum of BP patients were related to BP outcome (26). Moreover, anti-IL-17 therapy was revealed quite promising by using experimental murine and human-derived models of BP (27). However, these studies mostly concerned IL-17A and IL-17A/F isoforms, and to our knowledge, no study has evaluated the expression of the receptors of those cytokines yet, or the type of cells expressing those receptors in patients with BP.

IL-17 receptor family is composed of five members from IL-17RA to IL-17RE. The subunit IL-17RA is ubiquitous (28–31) and is a common co-receptor subunit for other members of the IL-17 family. IL-17RA pairs with IL-17RC to bind either IL-17A or IL-17F as covalent homodimers, but also IL-17A/IL-17F as heterodimers (32, 33). However, the association of IL-17RA with other members of the IL-17 receptor family shifts the affinity toward other members of the IL-17 family (34). Indeed, combination of IL-17RA with IL-17RB binds IL-17E as covalent homodimers (35–37). Besides, IL-17RA can also associate with IL-17RE to bind IL-17C as homodimers (38). However, the involvement of IL-17RA is not compulsory, as IL-17RB forms homodimers to target IL-17B as covalent homodimers (39). Up to now, no ligand for IL-17RD has been discovered yet. Also, the receptor of IL-17D remains unknown. In BP, IL-17A production has been mainly attributed to neutrophils, CD3^+^ T-lymphocytes, and mast cells (25, 27). Besides, IL-17RC expression was found increased in the skin of BP patients, whereas IL-17RA expression was unchanged (27). However, to better understand the role of IL-17 axis in BP, the pattern of IL-17 receptor isoform expression, and the cell types expressing those receptors both in the skin of BP patients and in the circulating immune blood cells need to be further investigated.

In this prospective study, we investigated the IL-17R isoform expression in immune blood cells of BP patients. We also analyzed the expression of IL-17 isoforms in the sera and in the BF of BP patients. To investigate the clinical involvement of the different IL-17 members in BP, we performed correlation studies between IL-17 concentration within the BF and the BPDAI (bullous pemphigoid disease activity index). Finally, to further delineate the role of IL-17 members in the inflammatory response within the BF associated with clinical activity of BP, we evaluated the expression of the respective IL-17 receptors at the surface of immune cells in lesional skin of patients.

## Materials and Methods

### Patients and Study Design

This prospective, single-center study was conducted between September 2013 and July 2017 in the Department of Dermatology at Reims University Hospital (French Referral Center for Autoimmune Bullous Diseases), under the approval of the Ethic Committee of the University Hospital of Reims (CNIL authorization DR-2013-320). In accordance with the Helsinki Declaration, all subjects gave their informed and written consent before their inclusion in the study. Patients that were included presented blistering skin dermatosis fulfilling at least three of four clinical criteria for BP according to Vaillant et al. (40), and in lesional skin, a basal lamina continuous deposit of IgG and/or C3 is revealed by direct immunofluorescence (IF) microscopy. Exclusion criteria were administration of a specific treatment for more than 2 days, pregnancy, and expected survival shorter than 3 months. Sera and blood cells were collected at different time points: at diagnosis (D0), 360 days after the diagnosis (D360), and at the time of a relapse (if it occurred). When possible, BF was collected at diagnosis (D0). Control samples were obtained from age- and sex-matched patients admitted to the department of traumatology, and orthopedic surgery of the same hospital. Controls that were included in the study did not have any autoimmune diseases and any clinical or biological signs of inflammation.

### Clinical Characteristics of BP Patients

Clinical data recorded at baseline were gender, age, and clinical activity of the disease evaluated by the Bullous Pemphigoid Disease Area Index (BPDAI) score (7). The BPDAI measures separate scores for mucous membrane and skin activities, the latter evaluating separately both cutaneous urticaria/erythema (non-bullous phase), and cutaneous blisters/erosions (blistering phase). As previously reported (41), a global BPDAI score ≥56 at baseline defined a severe disease while a global BPDAI score <56 defined a moderate disease.

### Cell Isolation

Peripheral blood mononuclear cells (PBMCs) and polymorphonuclear cells (PMNs) were isolated by density-gradient centrifugation from EDTA-treated whole blood (Granulosep, Eurobio-Abcys, France). Monocytes were then purified from PBMCs by means of positive selection with CD14 immunomagnetic beads (MACS; Miltenyi Biotec, Germany), according to the manufacturer's instructions. Then, flow through was used to isolate T lymphocytes by means of negative selection with the pan T-cell Isolation Kit from Miltenyi Biotec (MACS).

### IL-17R Gene Expression Analysis

Total RNA was extracted from isolated cells (3M, 5M, 7M, and 4M for monocytes, lymphocytes, PMNs, and PBMCs, respectively) using TRI-Reagent (Euromedex, Bas-Rhin, France) according to the manufacturer's protocol. cDNAs were synthetized and amplified using the Maxima First Strand cDNA kit with dsDNAse (Life Technologies), according to the manufacturer's instructions. The expression of IL-17RA, IL-17RB, IL-17RC, IL-17RD, IL-17RE, and β2-microglobulin (β_2_M) was analyzed by real-time quantitative PCR using the Power SYBR Green PCR Master Mix (Applied Biosystems) on the Stratagene Mx3005P (Agilent Technologies). Relative quantification was performed with β_2_M as a reference gene. Results were analyzed by using the MxPro QPCR software, and the fold regulation was calculated by using the 2^−ΔΔ*CT*^ method. Primer sequences (Eurofins) used to detect IL-17RA, IL-17RB, IL-17RC, IL-17RD, IL-17RE, and β_2_M are detailed in Table 1.

**Table 1:** Primer sequences used for amplification of IL-17R member and β2M genes by real-time quantitative PCR.

**Oligonucleotide**	**Forward sequence**	**Reverse sequence**
IL-17RA	TGCCCCTGTGGGTGTACTGGT	GCAGGCAGGCCATCGGTGTA
IL-17RB	TACCCCGAGAGCCGACCGTT	GGCATCTGCCCGGAGTACCCA
IL-17RC	CTGCCCTTGTGCAGTTTGG	CAGATTCGTACCTCACTCCCTA
IL-17RD	AGGCCTGGGTGAGGAGGAACC	GGGGAATCAGAGGGAGGCAGCA
IL-17RE	CCACCTTCAGGCCATGCAGCC	CTGTCATCCGTGTGGGAGGCC
β_2_M	ACCCCCACTGAAAAAGATGA	ATCTTCAAACCTCCATGATG

### Cytokine Measurement in Biological Fluids From BP Patients

IL-17A, IL-17A/F, IL-17B, IL-17C, and IL-17E were measured in control sera (*n* = 46), BP sera (*n* = 83), and BP BF (*n* = 31) using a U-PLEX assay (MesoScale Diagnostics; Rockville; USA). U-PLEX technology allows multiplex measurement of up to 10 cytokines within a single well in a volume of 50 μL. This technique is based on electro-chemiluminescence detection. Briefly biotinylated capture antibodies were coupled to U-PLEX linkers. The U-PLEX linkers then self-assembled onto unique spots on the U-PLEX plate. After binding to the capture antibodies, detection antibodies conjugated with electro-chemiluminescent labels (MSD GOLD SULFO-TAG) bound to the analytes to complete the sandwich immunoassay. The plate was then placed into an MSD instrument (SECTOR S6000 plate reader) to acquire data. Data analysis was performed by using MSD Workbench software. Limits of detection (LLOD) were 1.6 pg/ml for IL-17A, 3.0 pg/ml for IL-17A/F, 1.0 pg/ml for IL-17B, 3.0 pg/ml for IL-17C, and 0.76 pg/ml for IL-17E.

### IL-17R Detection in Isolated Monocytes From BP Patients

IL-17RA and IL-17RC expression was analyzed in monocytes isolated from BP patients and control subjects by immunocytochemistry (ICC). Isolated monocytes were cytospun and fixed with paraformaldehyde 4% (VWR). The primary antibodies rabbit anti-human IL-17RA (Bioss Antibodies, bs-2606R), and rabbit anti-human IL-17RC (Bioss Antibodies, bs-2607R) were applied to the cells and incubated overnight at 4°C. Chicken anti-rabbit IgG Alexa Fluor 594 (Life technologies, A21442) was used as secondary antibodies. Nuclei were stained with Hoechst 33342 (Thermofisher, Waltham, MA).

### IL-17R Detection in BP Skin Biopsy Specimen

Double IF staining were performed to analyze IL-17RA, IL-17RB, and IL-17RC expression on paraformaldehyde-fixed and paraffin-embedded lesional skin biopsy specimens from three BP patients before introduction of any treatment. All biopsies were obtained from the Pathology Department of Reims University Hospital. Briefly, after heat-induced antigen retrieval in Tris 10 mM EDTA buffer pH 9 and blocking in PBS 1×/BSA 3%, the primary antibodies rabbit anti-human IL-17RA (Bioss Antibodies, bs-2606R), rabbit anti-human IL-17RB (Gene Tex, GTX81729), rabbit anti-IL-17RC (Bioss Antibodies, bs-2607R), mouse anti-human mast cell tryptase clone AA1 (Dako, M7052), mouse anti-human CD163 (Novusbio, NB110-59935), and mouse anti-myeloperoxidase FITC (Abcam, ab11729) were applied and incubated overnight 4°C. Goat anti-mouse IgG Alexa Fluor 488 (Life technologies, A11029) and chicken anti-rabbit IgG Alexa Fluor 594 (Life Technologies, A21442) were used as secondary antibodies. Nuclei were stained with Hoechst 33342 (Thermofisher, Waltham, MA).

ICC and IF staining were visualized on an AxioObserver Z1 microscope (ZEISS) spinning disk ILAS 2 (Roper scientific). Image analysis was performed by using Metamorph software (Roper Scientific).

### Statistical Analysis

Statistical significance was inferred when necessary. GraphPad Prism 5 software (GraphPad, La Jolla, CA) was used for statistical analysis. Results are presented as mean ± SEM (standard error of the mean). Normality test was performed to evaluate Gaussian's distribution of the different population tested. We used non-parametric Mann–Whitney's test to compare two independent groups and non-parametric Wilcoxon's test to compare paired groups of population; chi-square test was used to evaluate qualitative variables. Pearson's correlation test was performed to explore the relationship between continuous variables. Results were considered significant when *p* < 0.05.

## Results

### Characteristics of the Studied Population

A total of 83 patients with BP were included in the study. The mean age at diagnosis was 81 years old and the sex ratio F/M was 1.9. A population of control subjects (*n* = 46) was used with no significant difference regarding these factors. At diagnosis, the total BPDAI score in the whole BP population was 40 ± 27 (Table 2). BP patients received superpotent topical corticosteroids as treatment, in association or not with systemic immunomodulators. Among the 83 patients included, 61 patients had a moderate BP, 22 patients a severe BP, 12 (14%) relapsed, 19 patients (24%) died, and 6 (7%) were lost during the year of follow-up. Baseline clinical characteristics of BP patient subgroups according to outcome are displayed in Table 2.

**Table 2:** Baseline clinical characteristics of patients with BP.

**(A) In the whole BP population included (*****n*** **=** **83)**			
Number of daily new blisters^*^	21 ± 36		
BPDAI total score^*^	40 ± 27		
BPDAI skin activity score^*^	38 ± 26		
- Blisters/erosions score^*^	25 ± 17		
- Erythema/urticaria score^*^	14 ± 14		
Patients with severe disease^a^, *n* (%)	22 (26)		
Patients with relapse^b^, *n* (%)	12 (14)		
**(B) In the subgroups of BP patients with (*****n*** **=** **12) and without relapse (*****n*** **=** **71)**			
	**BP without relapse**	**BP with relapse**	***p*****-value**
Number of daily new blisters^*^	21 ± 36	22 ± 32	0.2
BPDAI total score^*^	38 ± 27	49 ± 25	0.1
BPDAI skin activity score^*^	37 ± 27	47 ± 25	0.2
- Blisters/erosions score^*^	24 ± 17	32 ± 21	0.2
- Erythema/urticaria score^*^	14 ± 15	15 ± 12	0.5
**(C) In the subsets of BP patients with moderate (*****n*** **=** **61) and with severe disease (*****n*** **=** **22)**			
	**Moderate BP**	**Severe BP**	***p*****-value**
Number of daily new blisters^*^	9 ± 13	57 ± 52	0.0001
BPDAI total score^*^	26 ± 15	77 ± 17	0.0001
BPDAI skin activity score^*^	25 ± 15	75 ± 15	0.0001
- Blisters/erosions score^*^	17 ± 11	47 ± 13	0.0001
- Erythema/urticaria score^*^	9 ± 10	28 ± 16	0.0001

a *Severe disease was defined by BPDAI ≥ 56 (41)*.

b *Relapse was defined as the reappearance of at least three daily new blisters along with pruriginous, erythematous, or urticarial plaques*.

*Mann–Whitney's test was used for statistical analysis*.

* *Mean ± SD*.

### IL-17 Receptor Family Members in Immune Circulating Cells in BP

**Figure 1:**
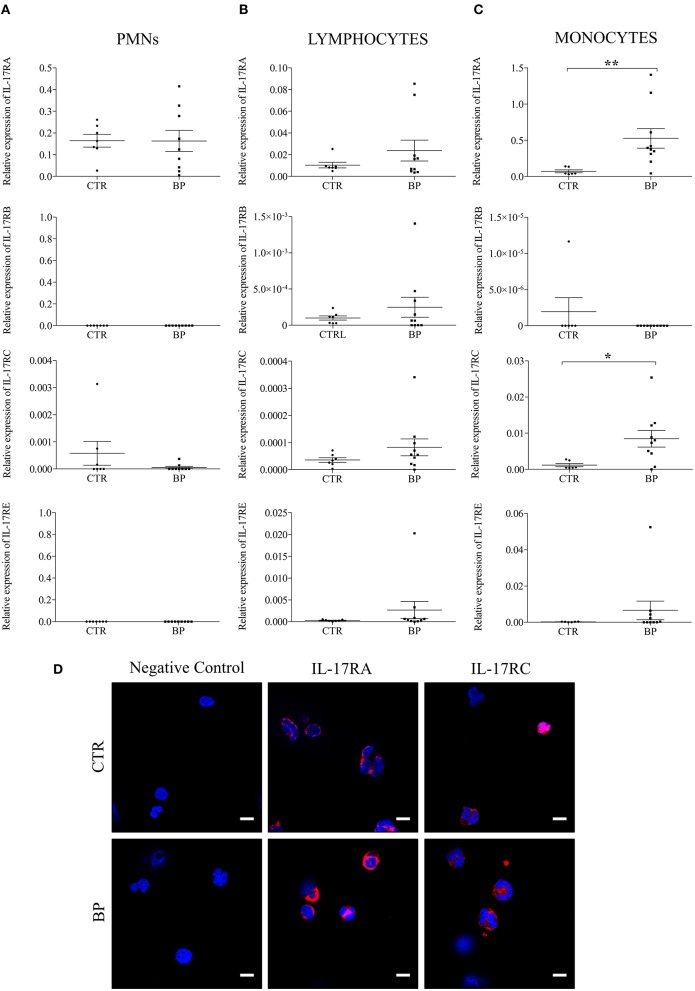
IL-17RA and IL-17RC mRNA expressions were increased in monocytes isolated from blood of patients with BP. The mRNA expression of IL-17RA, IL-17RB, IL-17RC, and IL-17RE were analyzed by real-time quantitative PCR in polymorphonuclear cells (PMNs) **(A)**, in lymphocytes **(B)**, and in monocytes **(C)** isolated from blood of control subjects (CTR), and of patients with BP at diagnosis (BP). Lines represent the mean, and non-parametric Mann–Whitney's test was used for statistical analysis (*p < 0.05; **p < 0.01). **(D)** Monocytes from BP patients and control subjects were stained for IL-17RA or IL-17RC. Negative control: primary antibodies were not added. Nuclei were counterstained with Hoechst (blue). Scale bar = 10 μm.

**Figure 2:**
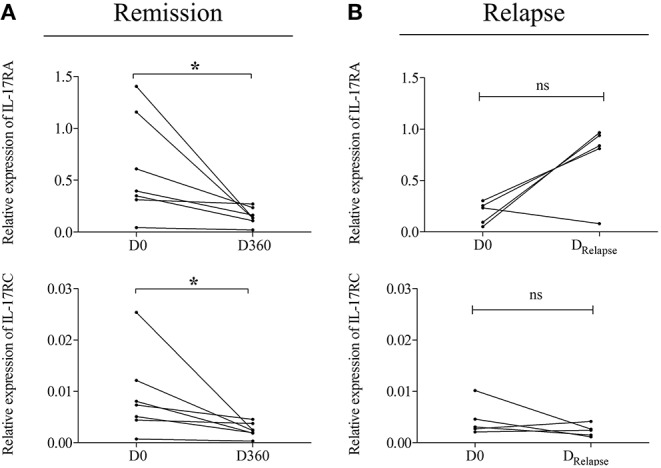
Analysis of IL-17RA and IL-17RC expression in monocytes issued from patients with BP according to disease outcome. IL-17RA and IL-17RC mRNA expressions were analyzed in monocytes of BP patients with ongoing remission **(A)** and of BP patients who relapsed **(B)**, at diagnosis (D_0_), and at day 360 (D_360_), or at the time of relapse (D_Relapse_), respectively. Non-parametric paired Wilcoxon's test was used for statistical analysis (*p < 0.05; ns = non-significant).

We first investigated the expression of each IL-17 receptor family member in circulating immune cells of patients with BP. At the time of diagnosis, circulating immune cells issued from BP patients or control subjects showed differential expression profile of each IL-17 receptor member. Indeed, whereas PMN cells mainly expressed IL-17RA mRNA (Figure 1A), lymphocytes expressed all IL-17 receptor members, except IL-17RD (data not shown), both in patients with BP and in control subjects (Figure 1B). In addition, in both lymphocytes and PMNs, no statistical differences could be observed at diagnosis between cells originated from BP patients and cells issued from control subjects. In contrast, a significant increase of IL-17RA, and IL-17RC mRNA expression (*p* = 0.006 and *p* = 0.016, respectively) was evidenced in monocytes from BP patients (*n* = 10) as compared with control subjects (*n* = 6) (Figure 1C). The overexpression of both receptors in monocytes from BP patients was also demonstrated at the protein level by IF staining (Figure 1D). Evaluation of the longitudinal variation of these receptors was realized on BP patients with an ongoing remission (*n* = 7), and in BP patients with relapse (*n* = 5) for whom blood samples were available. Baseline clinical characteristics in these subgroups of BP patient with and without relapse were similar to those displayed in Table 2 (data not shown). Both IL-17RA and IL-17RC mRNA increased expressions were significantly abolished after 1 year of treatment (D360) as compared to the level detected at the time of diagnosis (D0) (*p* = 0.016 and *p* = 0.016, respectively) in monocytes from patients with ongoing remission (Figure 2A). In contrast, in patients with relapse, both IL-17RA, and IL-17RC mRNA expression remained elevated at time of relapse (D_Relapse_). Indeed, no significant decrease of both IL-17RA and IL-17RC was demonstrated at time of relapse compared with diagnosis (Figure 2B).

### IL-17 Isoforms in Serum and BF of Patients With BP

**Figure 3:**
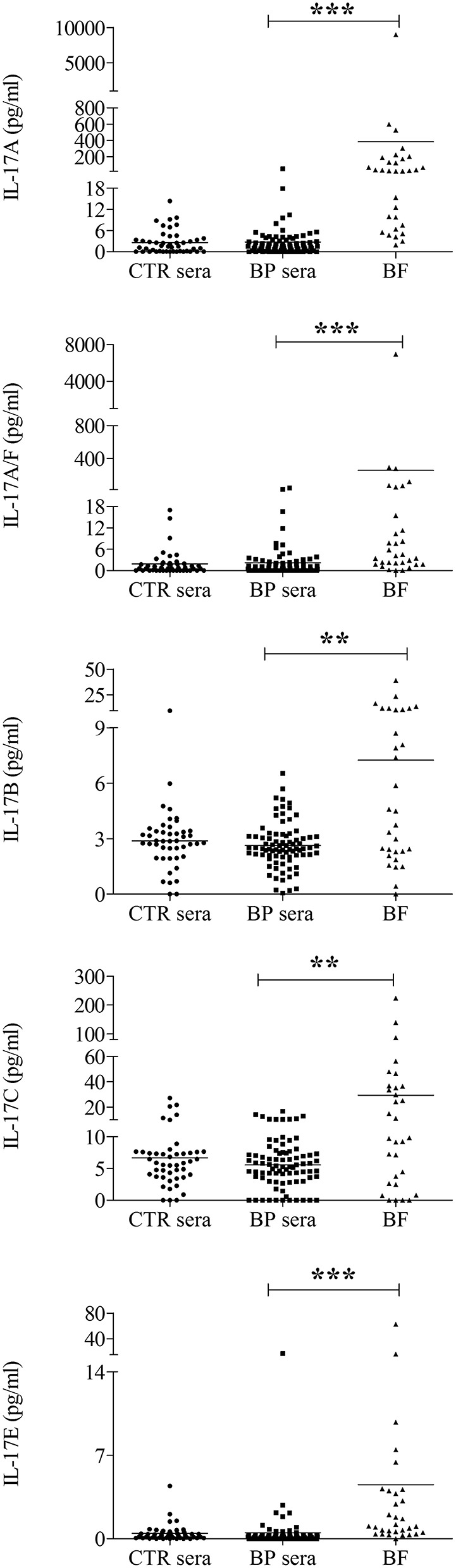
The expression of each IL-17 isoform tested was increased in blister fluid of BP patients. The concentrations of IL-17A, IL-17A/F, IL-17B, IL-17C, and IL-17E were measured at the time of diagnosis in serum (BP sera), and blister fluid (BF) of BP patients, and in serum of control subjects (CTR sera) using U-PLEX cytokine multiplex assay. Lines represent the mean, and non-parametric Mann–Whitney's test was used for statistical analysis (**p < 0.01; ***p < 0.001).

We next examined the expression of the IL-17 ligands including IL-17A, IL-17A/F, IL-17B, IL-17C, and IL-17E in biological fluids that could bind to the above-described IL-17R members expressed by circulating immune cells originated from BP patients. At the time of diagnosis, all tested IL-17 isoforms were detected in sera of control subjects (*n* = 46), and in sera (*n* = 83), and BF (*n* = 31) of patients with BP (Figure 3). Whatever the IL-17 isoforms analyzed, no significant differences were evidenced between sera from BP patients and sera from control subjects. In contrast, in BF of BP patients, the mean levels of each IL-17 isoform were all significantly increased as compared to BP sera concentrations (IL-17A, *p* < 0.0001; IL-17A/F, *p* < 0.0001; IL-17B, *p* < 0.01; IL-17C, *p* < 0.01; IL-17E, *p* < 0.0001) (Figure 3).

### IL-17 Isoforms and Disease Activity

**Figure 4:**
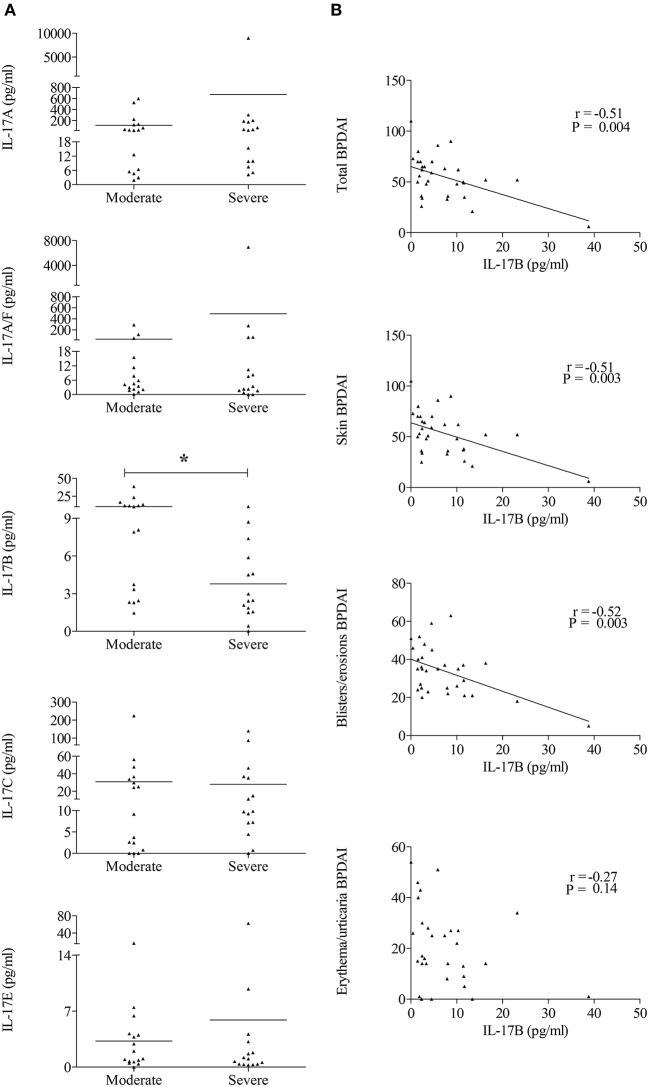
IL-17B levels were decreased in BP patients with severe disease extent and negatively correlated with blisters/erosions BPDAI subscore. **(A)** IL-17A, IL-17A/F, IL-17B, IL-17C, and IL-17E concentrations measured at the time of diagnosis in BF were analyzed according to the disease extent. “Moderate” stands for BP patients with a BPDAI <56 and “Severe” for BP patients with a BPDAI ≥56. Non-parametric Mann–Whitney's test was used for statistical analysis (*p < 0.05). **(B)** Correlations between total, skin, blisters/erosions, or erythema/urticaria BPDAI scores, and IL-17B levels in BF were analyzed using the Pearson's correlation test.

Investigation of whether the IL-17 isoform levels within the BF varied according to disease activity at diagnosis was performed on 16 BP patients with “mild to moderate” disease and on 15 BP patients with “severe” disease (Figure 4A). These subgroups were characterized by an increase of the total BPDAI score and skin subscores in BP patients with severe disease (BPDAI total score: 72 ± 14 vs. 39 ± 13, *p* = 0.0001; BPDAI skin activity subscore: 71 ± 14 vs. 38 ± 13, *p* = 0.0001; blisters/erosions subscore: 43 ± 11 vs. 26 ± 9, *p* = 0.0003; erythema/urticaria subscore: 29 ± 16 vs. 12 ± 10, *p* = 0.003 for severe and moderate BP patients, respectively). Figure 4A showed that IL-17B level was significantly higher (~3-fold, *p* = 0.01) in BF of BP patients with a mild to moderate disease as compared with levels measured in BF of BP patients with a severe disease. None of the other IL-17 isoforms showed significant variations according to disease extent. Deeper investigation revealed a significant and negative correlation between IL-17B and total BPDAI score (*n* = 31, *r* = −0.51, *p* = 0.004). Further analysis with respect to BPDAI subscores showed that IL-17B concentrations in BF were negatively correlated with the total skin BPDAI subscore (*n* = 31, *r* = −0.51, *p* = 0.003), and the skin blisters/erosions BPDAI subscore (*n* = 31, *r* = −0.52, *p* = 0.003) but not with the erythema/urticaria BPDAI subscore (*n* = 31, *r* = −0.27, *p* = 0.14) (Figure 4B).

### IL-17 Receptor Expression in Lesional Skin of BP Patients

**Figure 5:**
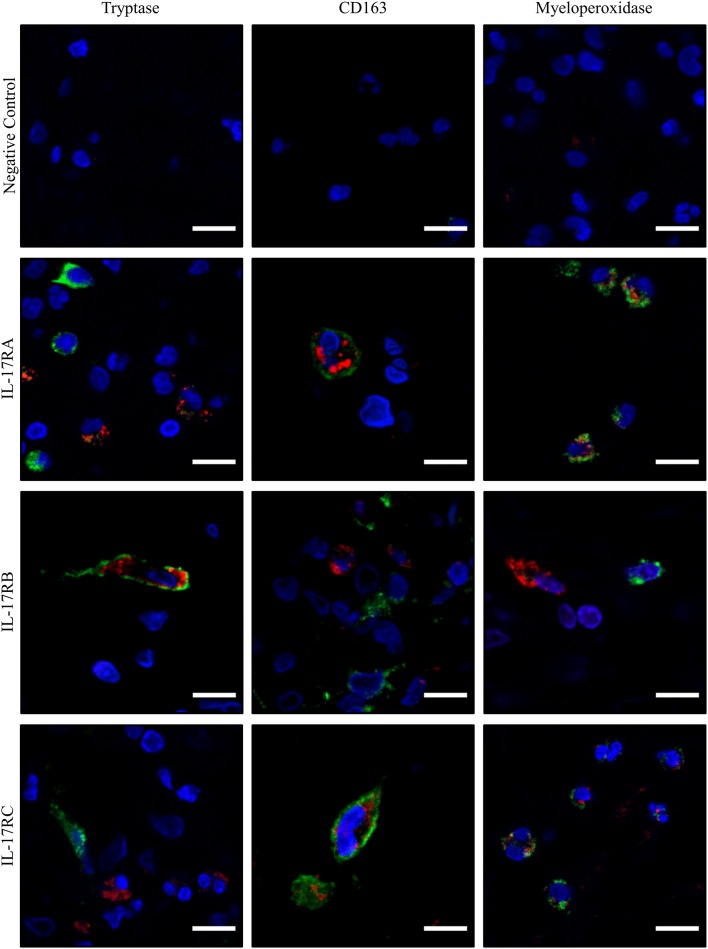
IL-17RA, IL-17RB, and IL-17RC showed differential pattern of expression in immune cells associated to lesional skin of patients with BP. Paraffin-embedded skin biopsy specimen of blistering skin from BP patients (*n* = 3) were subjected to double immunofluorescence staining for IL-17RA, IL-17RB, and IL-17RC (in red), and either Tryptase, CD163, or myeloperoxidase (MPO) (in green) with Hoechst counterstain. Negative control: primary antibodies were not added. Scale bar = 10 μm.

Finally, we wondered which cells within the blister cavity could be targeted by IL-17B. Double staining for IL-17RB and either mast cell (tryptase), macrophage (CD163), or neutrophil (myeloperoxidase, MPO) markers was performed in biopsies of BP patients (*n* = 3). Double staining revealed co-localization between IL-17RB and mast cell marker. Conversely, IL-17RB was not expressed by macrophages or neutrophils in blister cavity. In contrast, IL-17RA, and IL-17RC were expressed by both CD163^+^ macrophages and MPO^+^ neutrophils in blister cavity (Figure 5).

## Discussion

In this study, we demonstrated that IL-17 R expression displayed a peculiar pattern, with IL-17RA, and IL-17RC increased expression in monocyte and macrophages, whereas IL-17 RB was detected in mast cells. Noteworthy, IL-17RA and IL-17RC expression remained elevated in BP patients who relapsed under corticosteroid treatment. Furthermore, we also found that IL-17 A, B C, E, and F isoforms were all increased in the BF of BP patients. Although such IL-17 production suggested a broad involvement of the IL-17 axis in BP with active skin lesions before starting treatment, we only evidenced a negative correlation between BP disease activity and IL-17B, which could be mediated by IL-17RB expressed by mast cell in BP lesional skin.

We also showed that at the active phase of the disease and before treatment, IL-17RA and IL-17RC expressions were increased in monocyte, but neither in lymphocytes nor in neutrophils. Furthermore, no variation was observed in PBMCs (data not shown), suggesting that the overexpression is mainly restricted to monocytes. Actually, the critical role of monocytes in BP was already illustrated in a previous study showing that monocytes but not lymphocytes from BP patients responded to CXCL10 by increasing MMP-9 secretion (42). Thus, although all IL-17 receptors were detected in circulating blood cells, the overexpression of IL-17RA and IL-17RC in monocytes suggests that those receptors could play a critical role in BP. Actually, IL-17RA, and IL-17RC overexpression in monocytes could participate in BP relapse mechanisms as we observed that their expression was downregulated in BP patients whose disease was controlled by treatment, but remained overexpressed in BP patients who relapsed. Such results are in line with the IL-17 overexpression in the serum of BP patients with relapse under treatment (26) and with the capacity of those sera to increase NETosis in BP (43). This is also in setting with the fact that IL-17RC expression was found increased in the skin of BP patients (27), although in this study the authors found that IL-17RA expression remained unchanged. Indeed, in relation with the variations in monocytes, we also observed that IL-17RA and IL-17RC were markedly expressed by macrophages in the lesional skin of BP patients. Noteworthy, such IL-17RA, and IL-17RC expression in blister cavity was also demonstrated on neutrophils, further advocating on the role of both macrophages, and neutrophils in the dermal–epidermal cleavage upon IL-17 stimulation (25–27, 42, 43). Therefore, altogether, our results showed that investigation of both IL-17 isoforms and IL-17RA/RC may represent adequate tools to predict BP activity and outcome.

Here, we evidenced for the first time the expression of the different isoforms of IL-17 in the serum and in the BF of patients with BP. Furthermore, all of the IL-17 isoforms were produced at a higher level in the BF as compared to the serum of BP patients, suggesting a local production of these cytokines within the bullous area. However, the different IL-17 isoforms were produced at different levels. This is of importance as we also demonstrated that the expression of IL-17 receptors varied according to the immune cell types analyzed. Thus, this suggests that in BP, all combination of IL-17/IL-17R could be formed depending on the receptor types expressed at the surface of the cells, and on the affinity between these couples. IL-17A expression has already been mainly attributed to neutrophils, CD3^+^ T-lymphocytes, and mast cells (25, 27). The expression of other isoforms of IL-17 is much less defined. The expression of IL-17B and IL-17C has been attributed to T-cells (44). The expression of IL-17E has also been found to be produced by immune cells such as macrophage, lymphocyte, or mast cell (37, 45). Although the origin of the different IL-17 isoforms and of their receptors still needs to be further documented, the expression of all of those molecules of the IL-17 axis might conduct to a very intricate network in BP.

At baseline, only IL-17B expression was correlated with the BPDAI scores. Thus, we evidenced here, for the first time in BP, an IL-17 cytokine isoform capable of discriminating BP patients according to disease activity with the particularity that a negative correlation was observed between IL-17B and BP activity. Although further studies are required to demonstrate whether IL-17B expression is only a marker of disease activity or if this cytokine has a specific role in the regulation of the inflammatory response in BP, previous observation supports the idea that IL-17B could have a protective role in the process of inflammation (46). In BP, BPDAI subscore analysis showed that IL-17B was negatively correlated with the skin blisters/erosions lesions but not with the erythema/urticaria lesions. Therefore, IL-17B might not be involved in the pre-bullous phase of the disease. In contrast, the negative correlation between IL-17B and blisters and erosions lesions suggests that a BP patient with high IL-17B level would not form many blisters and erosions, and *vice versa*. IL-17B could interfere on BP pathogenesis by several mechanisms. On one hand, IL-17B could bind to IL-17RB, and scavenger at least in part IL-17RA to form the heterodimer IL-17RA/IL-17RB. This would reduce the pool of IL-17RA to enable to transduce the pathogenic effects of IL-17A and IL-17F either as homodimers or as heterodimers. In this way, this could limit the effects of IL-17E (IL-25) that bind to the IL-17RA/RB homodimer (46). On the other hand, binding of IL-17B onto its receptor could also transduce an anti-inflammatory response. Indeed, it has been shown that IL-17B is an anti-inflammatory cytokine (46). Given that IL-17RB is expressed by mucosal epithelial cells, such effects could be involved in the regulation of mucosal inflammation that occurs in certain patients with BP (47). However, why a patient would express a higher IL-17B than another patient is still unknown, and whether this level varies according to disease extent for a specific patient still needs to be investigated.

Besides, IL-17B concentrations within the BF were correlated neither with the anti-BP180NC16A nor with the anti-BP230 autoantibody titers (data not shown), suggesting that, in BP, IL-17B production was disconnected from the autoimmune process, which initiates blister formation. Also, no correlation was observed between serum and BF concentrations (data not shown), suggesting that IL-17B is mainly produced at the skin lesional site. Although we mentioned the pathogenic role of IL-17A or IL-17A/F above, we did not find any correlation with IL-17A or IL-17A/F with the BPDAI score and subscores. A potential explanation could be that the binding of an IL-17 isoform onto an IL-17 receptor may modulate the affinity and specificity of other IL-17 isoform receptor-binding event. This is of interest as this suggests that besides the IL-17RA pathways, other IL-17/IL-17R signaling cascades may be involved in the pathophysiology of BP. Noteworthy, IL-17RB also associates with IL-17RA to transduce the effects of IL-17E. In our study, we evidenced that the *in situ* expression of IL-17RB was attributed to mast cell within the blister cavity. This is in line with a previous study showing that mast cells expressed and responded to IL-17E, which also has IL-17RB as receptor (37). Thus, altogether, our results suggest that antagonist mechanisms may be involved in the formation of different IL-17 cytokine, and receptor complexes. However, further studies are required to better understand how the IL-17 network is related with clinical disease activity in BP.

Regarding the IL-17 cytokine family, up to now, the main focus was given to IL-17A and IL-17A/F in BP. In this study, our results promote the investigation of IL-17 RA and IL-17 RC as new relevant predicting factors in BP. Furthermore, we previously showed that IL-17A/F was a relevant marker of BP outcome (26), and a recent publication showed promising results in the use of anti-IL-17A by using both animal model of BP and a human-derived model of BP (27). Nevertheless, the therapeutic approach targeting the IL-17 axis should be seen from a broader angle. Actually, the IL-17RA isoform is ubiquitous and can combine with most of the other IL-17 receptor isoforms to conduct the message delivered by the different IL-17 isoforms. Indeed, we showed an increase of IL-17RA and IL-17RC at time of diagnosis of patients with BP. Thus, blocking the IL-17RA isoform would limit the effects of IL-17A and IL-17F. Although this still needs to be demonstrated, this could also limit the effects of other IL-17 isoforms that require the IL-17RA to transduce their effects such as the IL-17E, whose concentration is increased in the BF of BP patients. In contrast, this could favor the anti-inflammatory effects of IL-17B, which is independent of IL-17RA. Therefore, in BP like in other dermatologic diseases such as psoriasis, inhibiting IL-17RA may represent a good therapeutic option (48–50).

Taken together, the results of our study provide a better understanding of the pattern of IL-17 and IL-17 receptor isoforms in BP. In particular, this is the first study investigating the expression of IL-17 receptors by immune cells from patients with BP. We showed that, in BP, the expression of IL-17RA and IL-17RC in monocyte and macrophages are related to disease outcome, whereas IL-17B was negatively correlated to disease activity. Although further studies are required to demonstrate the role of the IL-17B/IL-17RB axis in the pathophysiology of this disease, our study highlighted an intricate IL-17 network with potential antagonist effects of these members of the IL-17 family.

## Data Availability

All datasets generated for this study are included in the manuscript/supplementary files.

## Ethics Statement

The studies involving human participants were reviewed and approved by Ethic Committee of the University Hospital of Reims (CNIL authorization DR-2013-320). The patients/participants provided their written informed consent to participate in this study.

## Author Contributions

PB, FA, and SL designed the study. SN, CM, and SL performed experiments. SN, CM, RL, PB, FA, and SL analyzed clinical and biological data. SN and SL performed the statistical analyses. CM, MV, and PB contributed to the clinical data collection. SN, RL, PB, FA, and SL wrote the manuscript. All authors critically evaluated the data and approved the final version for publication.

### Conflict of Interest Statement

The authors declare that the research was conducted in the absence of any commercial or financial relationships that could be construed as a potential conflict of interest.
